# Continuous-flow cryocompression therapy penetrates to bone level in hip fracture patients in a numerical simulation

**DOI:** 10.1186/s13018-019-1081-5

**Published:** 2019-02-14

**Authors:** Nick C. Leegwater, Sander M. van der Meer, Inger N. Sierevelt, Hugo Spruijt, Peter A. Nolte

**Affiliations:** 1 Department of Orthopedics, Spaarne Gasthuis, Spaarnepoort 1, 2134 TM Hoofddorp, The Netherlands; 2 Department of Clinical Physics, Spaarne Gasthuis, Hoofddorp, The Netherlands

**Keywords:** Cryotherapy, Hip fracture, Hypothermia, Analgesia, Thermodynamics

## Abstract

**Background:**

The aim of this study was to define deep tissue temperature during cryotherapy in postoperative hip fracture patients, by using measured skin temperature as input parameter for a simple numerical model. Second, an association was investigated between pain and tissue temperature distribution, to assess cryotherapy-induced analgesia of soft tissue-derived pain.

**Methods:**

Data from 35 participants in an ongoing trial was used. In three subjects who consented on optional measurements, skin temperature was measured in 3 days during and after cryotherapy. A simple numerical model was developed to calculate tissue temperature distribution during cryotherapy.

**Results:**

Inter and intrasubject skin temperature displayed high variation: trochanter 11–27 °C, mid-femur 11–24 °C, distal femur 10–16 °C. Predicted temperatures decreased to 20 °C at 1 cm, 26 °C at 2 cm, and 30 °C at 3 cm tissue depth. Smallest soft tissue layer was measured at the trochanter; 42% had less than 30 mm and 21% had less than 20 mm. Numeric rating scale pain varied (mean = 2.14; SD = 1.92), and no association was found between pain and decrease in temperature (*r* = 0.064; *p* = 0.204).

**Conclusions:**

Cryotherapy was predicted to reduce temperature up to 3 cm; in cachectic patients, this reaches the bone, where it might have implications for bone tissue healing when treated for a prolonged period of time. Cryotherapy-induced analgesia is likely to originate from skin analgesia rather than analgesia of muscle or bone-derived pain.

## Background

Cryotherapy is used to treat pain after musculoskeletal trauma such as total hip arthroplasty, total knee arthroplasty, and hip fractures [1–4]. Skin temperatures less than 13.6 °C produce skin analgesia [5]. In ankles, nerve conduction velocity is reduced with 33% at 10 °C skin temperature, resulting in a higher pain threshold [6]. Continuous-flow cryocompression therapy (CFCT) reduces skin temperature to 10 °C and to 22–25 °C up to 1.5 cm below the subcutaneous layer in the thighs of healthy individuals [7, 8]. Hypothermia diminishes the cell’s metabolic rate by two to fourfold per 10 °C drop in the mammalian central nervous system [9]. Mild hypothermia (28–34 °C) has shown to produce anti-inflammatory effects in healthy subjects [10], but sub-physiologic temperatures of less than 32 °C are also known to severely decrease proliferation in mammalian cells [11] and halts proliferation completely in mouse embryo fibroblasts [12]. In addition, at 34 °C hypothermia severely impairs osteoblast activity and doubles osteoclast activity in a calvarial rodent model [13]. Thus, a decrease in skin temperature is advantageous to treat pain, but adversely affects tissue metabolism, fibroblast, osteoblast, and osteoclast function that are needed for repair of soft tissue and bone. Whether cryotherapy reduces the temperature at the bone level after hip fracture surgery, possibly leading to impaired cellular function, is unknown. Furthermore, it is also unclear whether cooling of muscle and bone tissue at greater depth contributes to the analgesic effect of cryotherapy.

The aim of this study was to define deep tissue temperature during CFCT in postoperative hip fracture patients, by using measured skin temperature as input parameter for a simple numerical model. Second, an association between tissue temperature distribution and pain was investigated to assess cryotherapy-induced analgesia of soft tissue-derived pain. We hypothesized that (1) soft tissue temperature at the bone level (as determined on X-ray) will decrease during cryotherapy and (2) that temperature decrease and decline in pain perception by hip fracture patients as measured by numeric rating scale (NRS) pain are associated.

## Methods

The Medical Ethical Committee “METC Noord-Holland”, Alkmaar, The Netherlands (date: October 13, 2015; reference no: NH015.188) approved amendments in an ongoing trial that enabled additional measurements to be performed for the current study. These amendments also allowed subjects to be able to optionally consent with skin temperature measurements specifically for this study. After obtaining written informed consent, data on demographics, height, weight, implant type, pain scores before and after CFCT, and — if applicable — skin temperature measurements were obtained from subjects that participated in a multi-center trial [3]. All subjects that were allocated to receive CFCT at the Spaarne Gasthuis hospital as part of participation in this multi-center study were selected for data analysis in this study.

Continuous-flow cryocompression therapy was applied by using the “Game Ready System^®^” (GRS; CoolSystems Alameda, California) according to the cryotherapy treatment protocol as published earlier [14]. Through anatomically designed wraps, the GRS simultaneously delivers both adjustable continuous-flow cryotherapy and intermittent compression. The machine can be set at no pressure, low pressure (5–15 mmHg), medium pressure (5–50 mmHg), and high pressure (5–75 mmHg). Temperature can be adjusted between 4 °C and 13 °C; the lowest temperature was used. Pressure started at “low” and increased per four treatments to “medium” and “high.” Twelve treatment cycles were administered four times daily during the first 72 postoperative hours. A sole investigator applied and/or removed the wrap to perform the temperature measurements to reduce measurement variability. Before and after each treatment cycle, the subject was asked to verbalize perceived NRS pain.

In subjects who consented with optional skin temperature measurements, the cooling area was measured with a four-channel thermometer type TM-947SD with thermo probes type T; 12 M-T-0.5 Class 1 (Lutron Electronic Enterprise Co., Taipei, Taiwan) during and after CFCT on three consecutive evenings. Probes were placed laterally at the major trochanter, mid femur, the distal femur 5 cm proximal to the upper margin of the patella, and laterally on the contralateral femur. After probe placement, CFCT was administered for 30 min. Upon completion, the wrap was removed, and subjects were covered by normal blankets and remained in bed overnight. Skin temperature was measured every 10 s. For total hip arthroplasty, hemiarthroplasty and dynamic hip screw the mid femur temperature was used as input skin temperature for the model because most soft tissue trauma is present at this level, whereas the proximal skin temperature was used for the intramedullary hip nail since most soft tissue trauma is present at the entry point of the nail.

A simple validated numerical model was used to calculate tissue temperature during CFCT. The model uses a finite difference method to solve a one-dimensional temperature equation with the Crank-Nicolson scheme [15]. In order to solve the equation *Φ*(*x*,*t*) where *Φ* is the temperature, “*x*” is the skin depth, and “*t*” is time, the following three boundary conditions were used. First, the temperature at the skin (*x* = 0) equals the aforementioned measured skin temperature data (*Φ*(0,*t*) = *T*_meas_(*t*)). Second, the temperature at maximum depth “*L*” of 10 cm depth (*x* = *L*) equals the measured core temperature of the subject (*T*_0_) before CFCT (*Φ*(*L*,*t*) = *T*_0_). Finally, the initial temperature of the simulated body was also set to the measured core temperature of the subject before CFCT (*Φ*(*x*,*t*_0_) = *T*_0_). The body mass index (BMI) was calculated to determine the overall body mass. BMI and body fat percentage (BF%) are correlated, and based on this correlation, BF% was calculated and used as input parameter [16]. Assuming the tissue to be a homogeneous fat-water mixture, the thermal diffusivity was linearly interpolated between plain water (0.149 × 10^−6^ m^2^/s) and fat (0.1 × 10^−6^ m^2^/s), based on the BF% of the subject. A 1-s temperature resolution during a simulated time of half an hour and a spatial resolution of 0.1 mm were used.

**Fig. 1:**
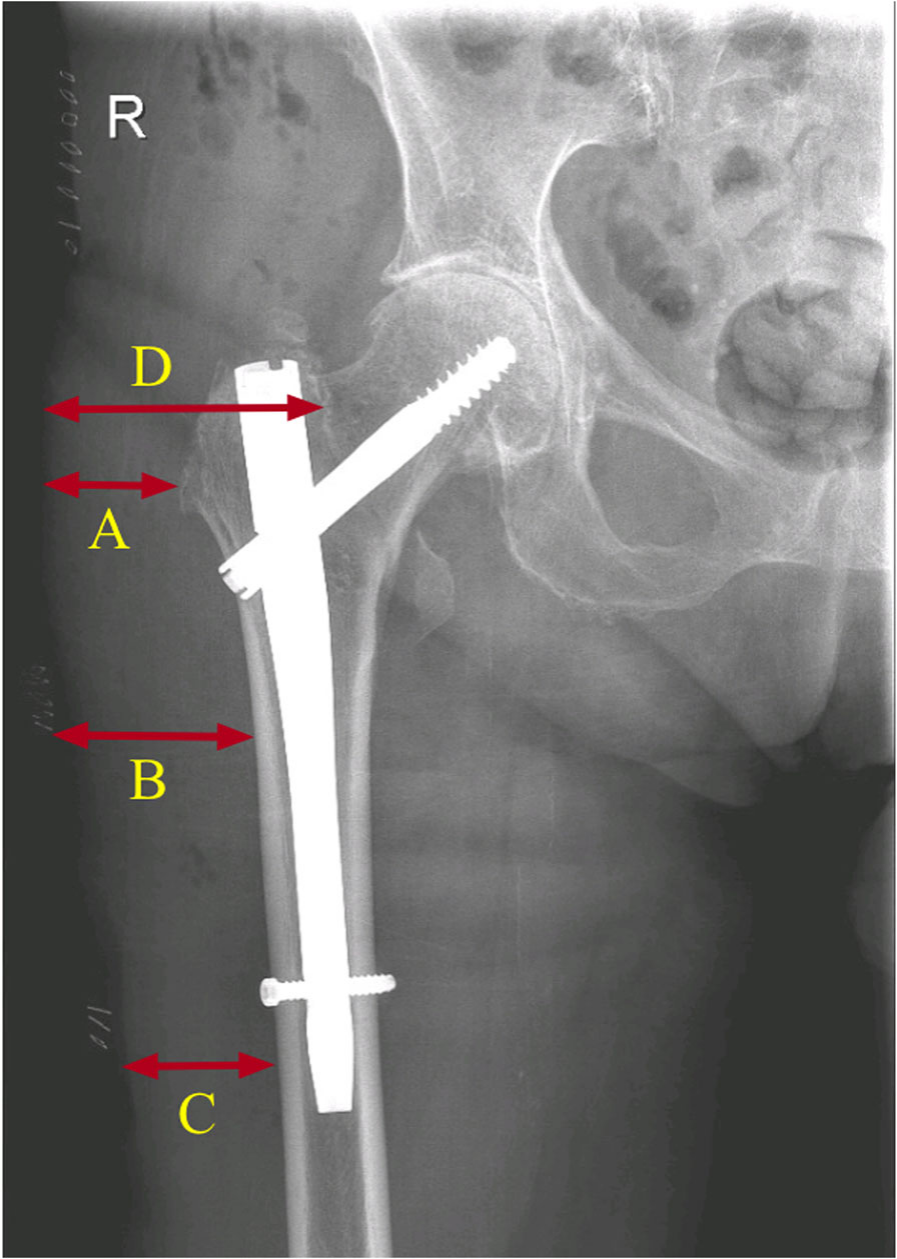
Tissue dimensions on an X-ray of an intramedullary hip nail for a right-sided peritrochanteric fracture. **a** Trochanter distance. **b** Mid femur distance. **c** Distal femur distance. **d** Shortest skin-to-fracture distance

Soft tissue dimensions were obtained from X-rays of all subjects (Fig. 1). The measurements were calibrated using implants with known dimensions. With the numerical simulation model, the minimum and mean temperature at those depths during CFCT was determined.

### Statistics

Statistical analysis was performed using IBM SPSS^®^ Statistics for Windows 25.0 (IBM, Armonk, NY). Both univariate and multivariate analyses were performed to assess the association between temperature distribution after CFCT and treatment effect during the first 72 postoperative hours. Treatment effect was defined as NRS pain improvement, which is defined as pretreatment NRS pain minus post-treatment NRS pain. Mixed model repeated measures analysis of covariance was performed and adjusted for potential confounders (gender, BMI, surgery type, fracture type, and pressure settings). Additional analysis was performed to assess the course of NRS pain after treatment during the first 72 h. A *p* value < 0.05 was considered statistically significant.

## Results

Data from 35 subjects were selected and eligible for data analysis; characteristics are displayed in Table 1. In ten subjects, incomplete pain measurements precluded pain analysis. Post-treatment NRS pain declined over the course of treatments during 72 h with 0.14 NRS per 6 h from 3.0 to 1.57 (*p* < 0.001), possibly reflecting reduction in inflammation and/or edema.

**Table 1:** Subject characteristics

Variable	Cryotherapy subjects (*n* = 35)
Female, *n*	26
Age, years	80.5 (40–95)
Weight, kg	66 (48–106)
Height, cm	168 (150–192)
BMI, kg/m^2^	22 (20–34)
Type of surgery, *n*	
DHS	9
HA/THA	12
IMHN	14

Data are reported as median and range unless stated otherwise

*BMI* body mass index, *DHS* dynamic hip screw, *HA* hemiarthroplasty, *THA* total hip arthroplasty, *IMHN* intramedullary hip nail

**Fig. 2:**
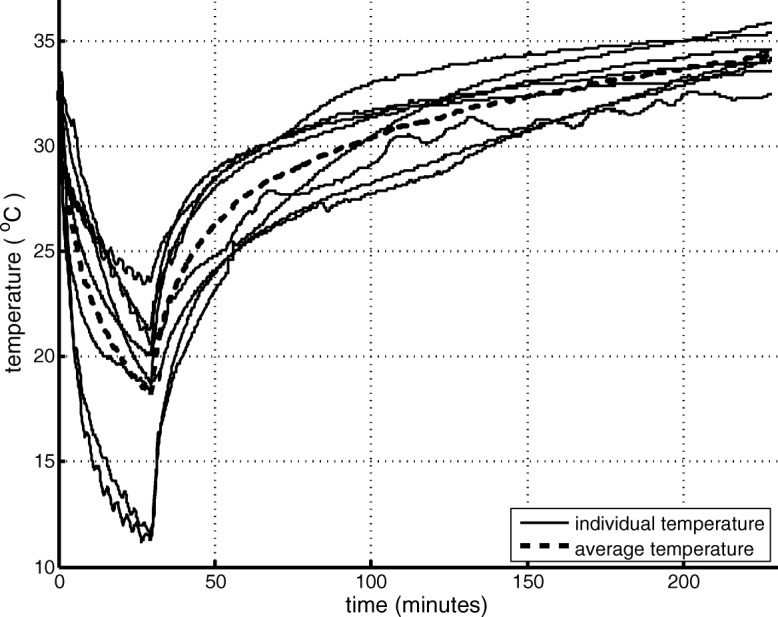
The average and individual temperature drop during 30 min of continuous-flow cryocompression therapy and subsequent passive rewarming measured at mid-femur

Three subjects consented with optional skin temperature measurements. Subjects 1 and 2 had a peritrochanteric fracture and an intramedullary hip nail was implanted, subject 1 was a cachectic male (age 76 years, weight 62 kg, height 182 cm), and subject 2 was an obese male (age 93 years, weight 100 kg, height 175 cm). Subject 3 was a cachectic female (age 91 years, weight 53 kg, height 150 cm) with a medial column fracture where a hemiarthroplasty was performed. In subject 1, only two registrations were obtained, and in one registration, two probes had a technical failure. In one registration, the distal temperature probe registered a minimum temperature of 30.8 °C, which was considered an error (probe was not covered by the wrap) and was omitted (Table 2). Inter and intrasubject skin temperature varied greatly. In subject 2, the high-pressure setting resulted in the lowest skin temperature measured, while in subject 3, the high-pressure setting did not result in a decrease in skin temperature. In all cases, the fastest decrease in skin temperature occurred the first 5 minutes of CFCT, and the minimum temperature reached was 11.5 °C at mid-femur after 27 min (Fig. 2). After cessation of CFCT, it took 5.5 min before the temperature exceeded 13.6 °C (the threshold that produces skin analgesia), and after 179 min (SD 52.7) baseline temperature was reached (Fig. 2). No reactive hyperthermia was observed after cessation of CFCT (Fig. 2).

**Table 2:** Skin temperature measurements during cryotherapy treatment

Subject no.	BMI	Timing	Pressure	NRS		Core temp. (°C)^‡^		Minimal skin temperature (°C)			
								Trochanter	Mid femur	Distal femur	Contralateral
				Pre	Post	Pre	Post				
1	18.7	POD 1	Low	–	–	–	–	–	–	–	–
		POD 2	High^*^	3	0	37.1	37.1	21	–	–	34
		POD 3	High	1	1	36.3	37.0	18	11.5	14	36
2	32.6	POD 1	Low	3	3	37.0	36.6	27	20	16	34.5
		POD 2	Medium	8	5	37.2	37.2	12	19	16	34
		POD 3	High	3	3	36.6	36.6	10.5	20.5	31	34
3	23.6	POD 1	Low	0	0	38.7	36.5	13	18.5	10	31
		POD 2	Medium	0	0	38.3	37.9	24	24	15	33.5
		POD 3	High	0	0	37.8	36.9	26	11.5	14.5	34.5

*POD* postoperative day, *NRS* numeric rating scale, 0–10

*Pressure was incorrectly set to high-pressure at day 2

^‡^Auricular measurement

**Fig. 3:**
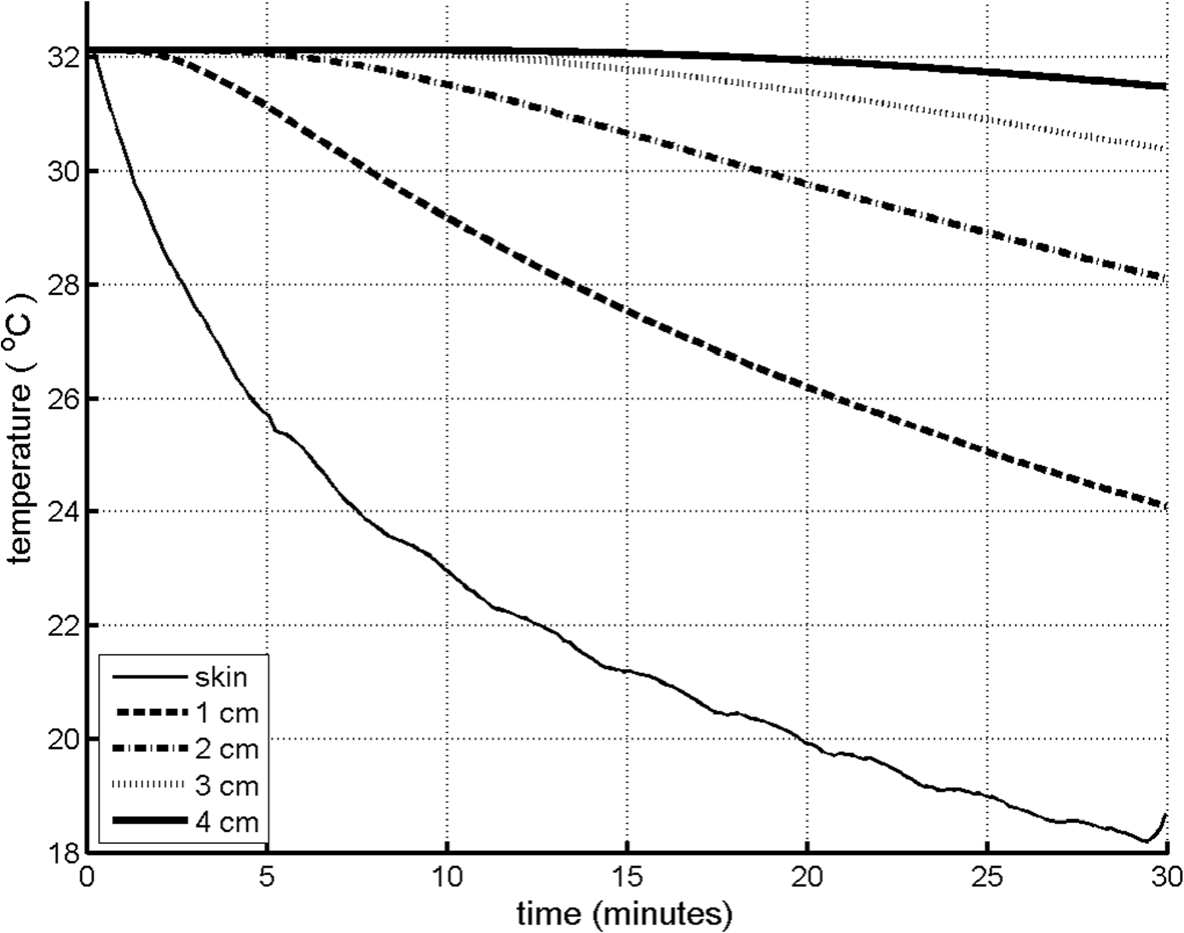
The average calculated temperature distribution during continuous-flow cryocompression therapy at various tissue depths

The initial measured skin temperature dropped on average from 32.1 °C to 18.2 °C after 30 min of CFCT. Consequently, in model simulations during CFCT (also up to 30 min), the average temperature dropped to 24.1 °C at 1 cm tissue depth, 28.1 °C at 2 cm tissue depth, and 30.4 °C at 3 cm tissue depth (Fig. 3). Temperature slightly dropped at 3 cm tissue depth and remained unaffected at deeper levels (Fig. 3). Soft tissue dimensions were acquired from postoperative X-rays in 24 subjects; in four subjects, no postoperative X-rays were taken; and in seven subjects, insufficient X-ray quality (soft-tissue reached outside the X-ray image) precluded measurements. Forty-two percent had a skin-to-bone distance of less than 30 mm, and 21% had a distance of 20 mm or less, the smallest usually being the trochanter (Fig. 1, arrow A). The lowest temperatures were observed at the trochanter and the distal femur (Table 3).

**Table 3:** Calculated deep soft tissue temperature after cryotherapy treatment

Dimension	X-ray* (mm)	Simulated temperature (°C) at X-ray distance		
		Minimal	Average	Maximal
A—Trochanter	34.1 (13.8)	27.1 (2.7)	30.1 (1.9)	31.9 (1.3)
B—Central	42.6 (13.6)	31.7 (1.7)	32.5 (1.1)	33.0 (0.7)
C—Distal	37.6 (11.6)	31.0 (2.0)	32.0 (1.3)	32.8 (0.8)
D—Fracture distance^A^	59.6 (10.8)	32.8 (2.1)	33.1 (1.3)	33.3 (0.8)

Data are reported as mean and standard deviation

*Skin distance based on first postoperative X-ray from 24 subjects

^A^The shortest distance measured from skin to the center of the fracture or the lateral confinement of the prosthesis in THA/HA subjects

No association was found between NRS pain and tissue temperature distribution at the central soft tissue (adjusted *β* coefficient 0.03 (− 0.24; 0.31) *p* = 0.81)).

## Discussion

Cryotherapy is used to alleviate pain and to reduce inflammation after musculoskeletal trauma [2, 17], but hypothermia might not always be beneficial for various cell types that are required for soft tissue and bone healing. It is important to understand to which depth CFCT reduces the temperature in order to put the effects on cellular function that are already known into perspective. Also, a correlation between tissue temperature distribution and pain perception might help to address the knowledge gap whether cooling of muscle and bone tissue at greater depth contributes to the analgesic effect of cryotherapy.

This is the first study attempting to define CFCT-induced deep tissue temperature drop and to correlate this decline with a change in pain in postoperative hip fracture patients. We found the minimum skin temperature to drop to 10–11 °C by cryotherapy. These observations are in line with others who measured a temperature of 12 °C on the skin at the distal thigh of healthy individuals with the same cryotherapy machine that was used in the current study [7]. Skin temperature drops to 10 °C when an ice pack or when a 4:1 water-alcohol (70%) mixture is applied [18]. The pressure setting did not affect cooling efficacy at the skin level in our obese subject, but it did augment cooling in our cachectic subject. In both ice-bag treatment and CFCT, external compression augments cooling efficacy [7, 8]. Although dynamic compression increases CFCT’s efficacy, it does not surpass the more traditional solid-state ice application modalities in skin temperature reduction. Heterogeneity in the application of the wrap can be an explanation for the varying skin temperatures. In addition, subjects with excess fat around the hips form the upper leg in a cone-like shape. This shape may cause difficulty in properly conforming the wrap to the thigh; the wrap usually covers most of the upper leg, whereas solid-state ice bags are applied directly to the target area.

In accordance with others [7, 8], in our model calculations, temperature dropped to 23 °C at 1.5 cm and to 26 °C at 2 cm tissue depth. Ice bag treatment combined with an elastic wrap reduces temperature to 25 °C at 2 cm in 30 min [8], whereas the cryotherapy device that was used in the current study reduces soft tissue to 23 °C at 1.5 cm depth at the high-pressure setting [7]. We found no reports on temperature drop at deeper levels. In our model, a temperature drop from 32.1 °C to 30.4 °C at 3 cm was the deepest level where a significant drop in temperature was calculated. Forty-two percent of the subjects had a skin-to-bone distance of less than 30 mm, and 21% had a distance of 20 mm or less at the major trochanter. This suggests that CFCT not only reduces soft tissue temperature but might also reduce bone temperature in hip fracture patients that have less than 30 mm of soft tissue. Temperatures such as 25–30 °C may retard connective tissue healing since proliferation is severely decreased [11], and proliferation halted in mouse embryo fibroblasts at 32 °C [12]. It might also have implications for bone healing, as osteoblast activity is severely impaired and osteoclast activity promoted in murine calvarial cells subjected to 34 °C [13]. We previously determined that hypothermia reduces vascular endothelial growth factor 165 (*VEGF-165*) protein expression under hypoxia, but we did not find the osteogenic differentiation of human adipose stem cells to be impaired [19]. Since the CFCT treatments in our study were applied intermittently for 30 min, the temperature drop is short-lived. However, if applied continuously and prolonged, CFCT might adversely affect connective and bone tissue healing.

Continuous-flow cryocompression therapy reduces pain in subjects that completed the 3-day treatment schedule [3]. In the current study, we attempted to determine an association between a predicted tissue temperature drop and a decline in NRS. However, we found no association between tissue temperature and pain perception. Two pathways can be proposed on how cryotherapy exerts its analgesic efficacy, either deeply via an interaction with tissue metabolism and immunomodulation or superficially via an interaction on nerve conduction. After induced soft tissue trauma, cryotherapy restores microcirculatory hemodynamics in rats [17]. Cryotherapy increases the level of anti-inflammatory cytokines IL-6 and IL-10, which are related to pain [20, 21] and decreases the pro-inflammatory Il-1α cytokine level in humans [10]. Regarding the superficial pathway, others determined skin temperatures of less than 13.6 °C to produce skin analgesia [5] and demonstrated reduced nerve conduction velocity due to decreased skin temperature [6]. The reduced nerve conduction velocity correlated with an increase in pain threshold, which was measured with a pressure algometer in the ankles of healthy subjects [6]. Objectively and reliably assessing patient-reported pain perception remains difficult; hence, standardized pain assessment methods are used. However, results obtained by these methods are difficult to translate to the clinical setting. It is questionable if this increase in pain threshold that is measured on the skin indeed provides a clinical noticeable analgesic effect in a postoperative setting, since postoperative pain originates from more than only skin trauma. In our subjects, skin temperature dropped below 13.6 °C, which should produce skin analgesia [5, 6] and the reduction of 1.5 NRS pain observed earlier [3] might illustrate this. However, the high variation in NRS pain and skin temperature we measured does hamper firm conclusions about whether the remaining pain originates from muscular tissue or the bone.

Some carefulness has to be taken when interpreting our findings. We did not measure skin folds although subjects with greater skinfold thickness required longer cryotherapy application time in order to produce similar tissue temperature changes [22]. Instead, we calculated the BF% from the BMI. The skin distances on the X-rays that were used in the model are static, and although edema or hematoma is not likely to dissipate within the first 72 h, it might be influenced by CFCT. Our model uses flat plate geometry; the simulated temperature drop at deeper distances will be underestimated. In reality, on the other hand, even though the therapy applies an external pressure to the tissue, the tissue will be perfused with a constant supply of warm blood. As this is not incorporated in the model, the model overestimates the temperature decrease. However, these effects act in opposite direction. With our simulation, we also found similar temperature and a similar penetration depths of 2–3 cm during 30 min CFCT, which matches to temperatures and penetration depths found in the literature [7, 8]. This suggests the simple model is adequate for our research questions.

## Conclusions

In our numerical simulation, we predicted cryotherapy to reduce temperature to approximately 3-cm tissue depth; in cachectic patients, this reaches the bone, where it might have implications for bone tissue healing when treated for a prolonged period of time. The lack of a correlation between pain and predicted decrease in temperature might implicate that cryotherapy-induced analgesia is likely to originate from skin analgesia rather than analgesia of muscle or bone-derived pain.

## Abbreviations

BF%: Body fat percentage
BMI: Body mass index
CFCT: Continuous-flow cryocompression therapy
GRS: Game Ready System
NRS: Numeric rating scale
VEGF: Vascular endothelial growth factor
